# Return of chloroquine-sensitive *Plasmodium falciparum* parasites and emergence of chloroquine-resistant *Plasmodium vivax* in Ethiopia

**DOI:** 10.1186/1475-2875-13-244

**Published:** 2014-06-25

**Authors:** Seleshi Kebede Mekonnen, Abraham Aseffa, Nega Berhe, Tilahun Teklehaymanot, Ronald M Clouse, Tamirat Gebru, Girmay Medhin, Thirumalaisamy P Velavan

**Affiliations:** 1 Aklilu Lemma Institute of Pathobiology, Addis Ababa University, Addis Ababa, Ethiopia; 2 Armauer Hansen Research Institute, Addis Ababa, Ethiopia; 3 Institute of Tropical Medicine, University of Tübingen, Tübingen, Germany; 4 Department of Medical Laboratory Science, College of Medical Sciences, Haramaya, Ethiopia; 5 Department of Bioinformatics and Genomics at University of North Carolina, Charlotte, North Carolina, USA; 6 Fondation Congolaise pour la Recherche Medicale, Brazzaville, Republic of Congo; 7 Addis Ababa University and Armauer Hansen Research Institute, Addis Ababa, Ethiopia

**Keywords:** Malaria, *Plasmodium falciparum*, *Plasmodium vivax*, Ethiopia, *pfcrt*, *pfmdr1*, *pvmdr1*, *pfmdr1* gene copy number

## Abstract

**Background:**

Increased resistance by *Plasmodium falciparum* parasites led to the withdrawal of the antimalarial drugs chloroquine and sulphadoxine-pyrimethamine in Ethiopia. Since 2004 artemether-lumefantrine has served to treat uncomplicated *P. falciparum* malaria. However, increasing reports on delayed parasite clearance to artemisinin opens up a new challenge in anti-malarial therapy. With the complete withdrawal of CQ for the treatment of *Plasmodium falciparum malaria*, this study assessed the evolution of CQ resistance by investigating the prevalence of mutant alleles in the *pfmdr1* and *pfcrt* genes in *P. falciparum* and *pvmdr1* gene in *Plasmodium vivax* in Southern and Eastern Ethiopia.

**Methods:**

Of the 1,416 febrile patients attending primary health facilities in Southern Ethiopia, 329 febrile patients positive for *P. falciparum* or *P. vivax* were recruited. Similarly of the 1,304 febrile patients from Eastern Ethiopia, 81 febrile patients positive for *P. falciparum* or *P. vivax* were included in the study. Of the 410 finger prick blood samples collected from malaria patients, we used direct sequencing to investigate the prevalence of mutations in *pfcrt* and *pfmdr1*. This included determining the gene copy number in *pfmdr1* in 195 *P. falciparum* clinical isolates, and mutations in the *pvmdr1* locus in 215 *P. vivax* clinical isolates.

**Results:**

The *pfcrt* K76 CQ-sensitive allele was observed in 84.1% of the investigated *P.falciparum* clinical isolates. The *pfcrt* double mutations (K76T and C72S) were observed less than 3%. The *pfcrt* SVMNT haplotype was also found to be present in clinical isolates from Ethiopia. The *pfcrt* CVMNK-sensitive haplotypes were frequently observed (95.9%). The *pfmdr1* mutation N86Y was observed only in 14.9% compared to 85.1% of the clinical isolates that carried sensitive alleles. Also, the sensitive *pfmdr1* Y184 allele was more common, in 94.9% of clinical isolates. None of the investigated *P. falciparum* clinical isolates carried S1034C, N1042D and D1246Y *pfmdr1* polymorphisms. All investigated *P. falciparum* clinical isolates from Southern and Eastern Ethiopia carried only a single copy of the mutant *pfmdr1* gene.

**Conclusion:**

The study reports for the first time the return of chloroquine sensitive *P. falciparum* in Ethiopia. These findings support the rationale for the use of CQ-based combination drugs as a possible future alternative.

## Background

Malaria remains one of the major health problems in sub-Saharan Africa [1,2]. Though there are encouraging reports that malaria morbidity and mortality are declining [3], it is still an overwhelming public health problem, with an estimated 207 million cases and 627,000 deaths every year worldwide. Of all malaria deaths, 90% occur in sub-Saharan Africa, and 77% in children under five years of age [4]. One of the main obstacles to malaria control is the ability of the parasites to develop resistance to the administered anti-malarial drugs. Chloroquine (CQ) was the first antimalarial to be widely used in endemic areas, but CQ resistance was documented in Thailand in the late 1950’s, spread to African in 1974 [5], and subsequently came to East Africa in the early 1980’s [6]. Since 2009, there are increasing concerns and reports of delayed parasite clearance to administered artemisinin, especially in parts of Southeast Asia [7].

In Ethiopia, CQ treatment failure for *Plasmodium falciparum* and *Plasmodium vivax* was documented in 1996 [8]. Therefore, in 1998 sulphadoxine-pyrimethamine (SP) replaced the CQ triple-dose (25 mg base/kg) regimen as the first-line drug for the treatment of uncomplicated *P. falciparum* and mixed infection of *P. falciparum and P.vivax* malaria. However, in a short time span *P. falciparum* also developed resistance to SP, leading to therapeutic failure of SP [9-12]. Artemether-lumefantrine (AL) (Coartem®) replaced SP in 2004 as a first-line drug to treat uncomplicated *P. falciparum* and mixed infection of *P. falciparum and P.vivax* malaria in Ethiopia [13]. However CQ still remains the first-line drug to treat *P. vivax* malaria in Ethiopia and sub-Saharan Africa. Although significant progress has been made to understand the resistance mechanisms to administered anti-malarial drugs, the genetic basis of drug resistance is vital to implement strategies for efficient malaria control.

Point mutations in the genes “*P. falciparum* multi-drug resistance 1” (*pfmdr1*), located in chromosome 5 [14-17], “*P. falciparum* chloroquine resistance transporter” (*pfcrt*), on chromosome 7 [18-20], and “*P. vivax* multi-drug resistance 1” (*pvmdr1*) on chromosome 10 [21-23] are the most investigated genes for anti-malarial drug resistance. *Pfmdr1* encodes a trans-membrane transporter, and point mutations in this gene modulate the level of CQ resistance [24,25]. Other mutations are also associated with anti-malarial drug resistance and delayed parasite clearance [15,17,26,27]: in the codons 86 (Asn86Tyr), 184 (Tyr184Phe), 1034 (Ser1034Cys), 1042 (Asn1042Asp), 1246 (Asp1246Tyr) in *pfmdr1*; codons 72 (Cys72Ser), 74 (Met74Ile), 75 (Asn75Glu) and 76 (Lys76Thr) in *pfcrt*; and codons 976 (Tyr976Phe) and 1076 (Phe1076Leu) in *pvmdr1*. Additionally, several other studies have also reported that *pfcrt* haplotypes are associated with anti-malarial drug resistance [19,25,28]. Gene copy number variations (CNV) in the *P. falciparum* genome have been shown to influence parasite phenotypes, and increased gene copy numbers are associated with reduced susceptibility to anti-malarial drugs.

Mutations in the *pfmdr1* locus are associated with anti-malarial resistance to chloroquine, mefloquine, quinine or artemisinin derivatives, whereas *pfcrt* mutations are associated with chloroquine, amodiaquine, mefloquine and artemisinin combination therapy [18]. Increased pfmdr1 gene copy numbers are associated with reduced susceptibility of P. falciparum to anti-malarial drugs, especially with artemether-lumefantrine and artesunate-mefloquine [29]. In many countries that are prone to malaria endemics, *P. vivax* malaria is treated with CQ, as the drug is well tolerated, affordable, has a longer half-life, and protects from early relapses [30-32]. A decline in CQ efficacy for *P. vivax* malaria was reported in Papua New Guinea in 1989 [33] and in northwestern Sumatra, Indonesia in 1995 [34]. CQ resistance in *P. vivax* in Ethiopia was reported in 2008, when four patients out of 83(4.8%) had recurrent parasitaemia on Day 28 [35].

Several studies at regular intervals are required to evaluate drug resistance based on the genetic diversity of circulating *P. falciparum* strains and their molecular markers for *P. falciparum* resistance to CQ and AL. Such studies will lay a basis to re-evaluate existing strategies in malaria control [36]. In the present study, the prevalence of mutations in *pfcrt* and *pfmdr1*, the gene copy number of *pfmdr1* in *P. falciparum*, and point mutations in *pvmdr1* in *P. vivax* were investigated in clinical isolates from Southern and Eastern Ethiopia.

## Methods

### Study sites

A health institution-based study targeting febrile patients attending primary health facilities was conducted in Southern and Eastern Ethiopia. Samples from Southern Ethiopia were collected between the months of August and December, 2011, at three health centres: Omo Nada, Bala Wajo, and Arba Minch. In Eastern Ethiopia samples were collected in the Harar health centre between the months of October and December 2009. Malaria transmission in these study sites is seasonal, with higher transmission following the rainy seasons (between September and December, and between April and May).

### Study samples

Study participants were recruited from the routine health delivery system. Self-presenting febrile patients attending health centers in Omo Nada (n = 713), Bala Wajo (n = 312), and Arba Minch (n = 391), and whose age was at least six months between August and December 2011, were eligible for the current study. Additionally self-presenting febrile patients attending Harar health centre (n = 1,304) in eastern Ethiopia were also eligible for the study. An axillary temperature was measured to check for a fever. Patients with an axillary temperature of ≥37°C were considered febrile. Any patient reported as being infected either with *P. falciparum* or *P. vivax* or with a mixed infection using the existing health care delivery system within the target health centres were approached for consent immediately after receiving their laboratory result. For those who consented to be part of the study, approximately 5–10 μL of a finger-prick blood sample was collected. No additional clinical assessment was carried out at the time of recruitment. A total of 410 Southern and Eastern Ethiopian finger-prick blood samples collected on Whatman 3 mm filter paper tested positive by microscopy for either *P. falciparum* or *vivax*. Of these, 195 tested positive for *P. falciparum* (170 from Southern Ethiopia and 25 from Eastern Ethiopia), and 215 for *P. vivax* (159 *P. vivax* from Southern Ethiopia and 56 from Eastern Ethiopia). The blood spots were air-dried and stored in a separate, clean, sealed plastic bag at room temperature for further molecular analysis.

### Blood film management

After fixing the thin film in methanol, both smears were stained with 3% Giemsa for 30 min and examined under oil immersion for malaria parasites. Thick films were used for parasite detection, and thin film for species identification. At least 100 fields examined under an oil-immersion lens were examined before ruling out infection.

### Detection of *pfmdr1*, *pfcrt* and *pvmdr1* polymorphisms

DNA was isolated using the Qiagen DNA Mini Kit for blood and tissue (QIAGEN, Germany), following the manufacturer’s instructions, and it was then stored at −20°C until use. Nested PCR was carried out as previously described elsewhere [37]. DNA samples were amplified by species-specific primer pairs (Table 1). For *pfcrt, pfmdr1* and *pvmdr1* both the primary and nested amplifications were carried out in a 20 μL reaction volume containing 1X buffer, 2.5 mM MgCl_2_, 200 μM dNTPs, 200nM primers, 1U Taq DNA-Polymerase, and 10 ng of DNA template on a PTC-200 Thermal cycler (MJ Research, USA). Each genus-specific amplification for *Plasmodium* was followed by a species-specific PCR. For amplifications, the study followed standard nested PCR procedure (Table 1). The PCR product of the first reaction was used as the template for the second in all nested PCRs. Amplicons that resulted from the nested PCRs were separated by electrophoresis on a 1.2% agarose gel, run with a 100 bp DNA ladder (Invitrogen, Karlsruhe, Germany). The presence of different *Plasmodium* species was re-confirmed by checking amplicon size. To identify relevant Single Nucleotide Polymorphisms (SNPs) in *pfmdr1, pfcrt* and *pvmdr1*, PCR products were cleaned using Exo-SAP-IT (USB, Affymetrix, USA), and 1 μL of the purified product was used as a template for direct sequencing using the Big Dye terminator v. 2.0 cycle sequencing kit (Applied Biosystems, USA) on an ABI 3130XL DNA sequencer, according to the manufacturer’s instructions [38]. All investigated point mutations in *pfmdr1, pfcrt and pvmdr1* are illustrated in Table 2.

**Table 1:** **Primer pairs used to amplify and sequence *pfcrt, pfmdr1 *** **and *pvmdr1***

**Gene fragment**	**Primer Name**	**Primer sequence**	**PCR cycling conditions**
Pfcrt SNPs at	OF P1	5′CCGTTAATAATAAATACACGCG	35 cycles of 94°C for 30 s; 56°C
Codon 72&76	OR P2	5′CGGATGTTACAAAACTATAGTCC	for 30 s; and 62°C for 1 min;
	NF P3	5′-AGGTTCTTGTCTTGGTAAATTTGC	30 cycles of 94°C for 30 s; 56°C
	NR P4	5′-CAAAACTATAGTTACCAATTTTG	for 30 s; and 65°C for 1 min;
Pfmdr1 SNPs at	OF P5	5′-AGGTTGAAAAAGAGTTGAAC	30 cycles of 94°C for 30 s; 55°C
Codon 86&184	OR P6	5′-ATGACACCACAAACATAAAT	for 30 s; and 65°C for 1 min;
	NF P7	5′-ACAAAAAGAGTACCGCTGAAT	30 cycles of 94°C 30 s; 60°C
	NR P8	5′-AAACGCAAGTAATACATAAAGTC	for 30 s; and 65°C for 1 min;
Pfmdr1 SNPs at	OF P9	5′-GTGTATTTGCTGTAAGAGCT	34 cycles of 94°C for 30 s; 55°C
Codon 1034	OR P10	5′-GACATATTAAATAACATGGGTTC	for 1 min and 72°C for 1.5
1042 and 1246	NF P11	5′ CAGATGATGAAATGTTTAAAGATC	29 cycles of 94°C for 30 s; 60°C
	NR 12	5′-TAAATAACATGGGTTCTTGACT	for 30 s; and 65°C for 1 min;
pvmdr1at codon	OF P13	5′-GCGAACTCGAATAAGTACTCCCTCTA	45 cycles of 94°C for 5 min,
976 and 1076	OF P14	5′GGCGTAGCTTCCCGTAAATAAA	530C for 1 min and 720C for 1 min
	NF P15	5′-GGATTGCTGTCAGCACATATTAACA	45 cycles of 94°C for 5 min,
	NF P16	5′AGAGGGATTTCATAAAGTCATT	650C for 1 min and 720C for 1 min

OF: Outer Forward; OR: Outer Nested; P1-P12: Primers; NF: Nested Forward; NR: Nested Reverse; bp: base pair; pfmdr1: *Plasmodium falciparum* multi-drug resistance 1; pfcrt: *Plasmodium falciparum* chloroquine resistance transporter; pvmdr1: *Plasmodium. vivax multi-drug resistance 1.*

**Table 2:** **Investigated point mutations in *pfmdr1, pfcrt *** **and *pvmdr1*** **genes**

			***Pfmdr1 *** **locus**				**Pfcrt locus**			***Pvmdr1 *** **locus**	
Codon position	86	184	1034	1042	1246	72	74	75	76	976	1076
Wild type/Mutant	TGA/TG***T***	ATA/AT***T***	CAG/C***T***G	TTA/TT***G***	GAG/GA***T***	TGT/***A***GT	ATG/AT***T***	AAT/***G***A***A***	AAA/A***C***A	TAC/T***T***C	TTT/***C***TT
ns substitution	Asn/Tyr	Tyr/Phe	Ser/Cys	Asn/Asp	Asp/Tyr	Cys/Ser	Met/Ile	Asn/Glu	Lys/Thr	Tyr/Phe	Phe/Leu

ns: non synonymous ; *pfmdr1*: *Plasmodium falciparum* multi-drug resistance 1; *pfcrt*: *Plasmodium falciparum* chloroquine resistance transporter; *pvmdr1*: *Plasmodium vivax* multi-drug resistance 1.

### Detection of *pfmdr1* gene copy number variations

The *pfmdr1* gene copy number was estimated by TaqMan real-time PCR, as previously described [29]. Briefly, 10 ng of genomic DNA was amplified in a 25 μL volume of reaction mixture containing 1x TaqMan buffer (8% glycerol, 0.625 U DNA polymerase, 5.5 mmol/L MgCl2, 300 μmol/L dNTPs, 600 nmol/L passive reference dye ROX (5-carboxy-X-rhodamine), pH 8.3), 300 nmol/L of each forward and reverse primer, 100 nmol/L of each probe, and 5 μL of template DNA on a Corbett Research RG-3000 (Qiagen, Hilden, Germany). Thermal cycling parameters were as follows: pre-incubation at 95°C for 5 minutes, followed by 50 cycles of 15 seconds at 95°C and 1 minute at 58°C. Genomic DNA from *P. falciparum* reference 3D7, reported to have only one copy, was used as the calibrator [39], and *P. falciparum* ß-tubulin as the house-keeping gene. For multiple *pfmdr1* copy number control, DNA from the Dd2 clone was used. The 2^-ΔΔCt^ method of relative quantification [40] was used to estimate copy number. Using this cycle threshold (Ct) method to estimate copy numbers of unknown samples, a calibrator DNA template with known copies of the interest and a house-keeping gene which has the same copy number was utilized. The ΔΔCt calculation was as follows: ΔΔCt = (Ct target gene - Ct Pf ß-tubulin) X - (Ct target gene - Ct Pf ß-tubulin) Y, where X = unknown sample and Y = *P. falciparum* 3D7. The result was expressed in N-fold changes in X target gene copies normalized to *P. falciparum* ß-tubulin according to the equation (amount of target) = 2^-ΔΔCt^. Each sample was run as triplicate, and the mean and standard deviation of the three Ct values were calculated and computed for specificity as suggested earlier [29].

### Ethics

Ethical approvals were obtained from the review boards of Aklilu Lemma Institute of Pathobiology (ALIPB), College of Health Sciences of Jimma University, College of Health and Medical Sciences of Haramaya University and Armauer Hansen Research Institute (AHRI). After obtaining informed consent and assent from either the patient or legal guardian, blood samples were collected from individuals positive for *P. falciparum* or *P. vivax*. The study participation was voluntary and had no influence on the treatment provision at the respective health facilities. All patient information was kept confidential. All individuals positive for *P. falciparum* were treated with AL, while *P. vivax* positive patients were treated with CQ.

## Results

The observed frequencies of the investigated SNPs in the *pfmdr1, pfcrt* and the *pvmdr1* loci are summarized in Table 3. The *pfcrt* K76T (Lys76Thr) mutation was observed in only 15.9% (31/195) of the clinical isolates investigated compared to 84.1% (164/195) wild type allele that were sensitive to CQ. The *pfcrt* C72S (Cys72Ser) was observed only in 3.6% (7/195) compared to 96.4% (188/195) wild type allele that were sensitive to CQ. The *pfcrt* double mutations (K76T and C72S) were observed in less than 2.6% of individuals (5/195) Table 3. For the investigated *pfmdr1* locus N86Y(Asn86Tyr), the mutation was observed in 14.9% (29/195) of the clinical isolates compared to 85.1% (166/195) wild type that carried sensitive alleles. The investigated *pfmdr1* locus Y184 (Tyr184Phe), only 5.1% (10/195) of the clinical isolates carried the mutant. None of the investigated 195 *P. falciparum* clinical isolates carried S1034C, N1042D and D1246Y of *pfmdr1* polymorphisms. For the investigated *pvmdr1* locus Y976 (Tyr976Phe), the mutation was observed in 32.6% (70/215) of the clinical isolates compared to 67.7% (145/215) wild type that carried sensitive alleles, whereas the investigated F1076 (Phe1076Leu) carried no drug resistant mutants Table 3. Of the all investigated *P. falciparum* clinical isolates from Southern and Eastern Ethiopia, only one copy of *pfmdr1* gene copy number was observed.

**Table 3:** **Distribution of *pfcrt*, *pfmdr1 *** **and *pvmdr1 *** **point mutations in the investigated clinical isolates**

	***P. falciparum***	**number**	**%**
	**no resistance alleles detected**	**127**	**65**%
*Pfcrt*	only C72	7	3.6%
	C72 and K76	5	2.6%
	only K76	31	15.9%
*pfcrt* and *pfmdr1*	K76 and N86	4	2.1%
*pfmdr1*	only N86	29	14.9%
	N86 and Y184	8	4.1%
	only Y184	10	5.1%
	Total	195	
	*P. vivax*		
	**no resistance alleles detected**	**145**	**74**%
*pvmdr1*	only Y976	70	32.6%
	only F1076	0	0%
	Total	145	

The frequency of SNPs in the *pfmdr1, pfcrt* and the *pvmdr1* loci in Southern and Eastern Ethiopia are summarized in Table 4. The *pfcrt* K76T mutation in Southern Ethiopia was 13.5% (23/170) of the clinical isolates investigated compared to 86.5% (147/170) wild type allele that were sensitive to CQ. The *pfmdr1* locus N86Y was observed in 12.9% (22/170) compared to 87.1% (148/170) wild type allele that were sensitive to CQ. In Eastern Ethiopia, SNPs in the *pfmdr1* locus N86Y and *pfcrt* locus K76T were 28.0% (7/25) and 32.0% (8/25) respectively. On the other hand, for the investigated *pvmdr1* locus Y976 (Tyr976Phe), the mutation was observed in 37.1% (59/159) of the clinical isolates in Southern Ethiopia compared to 19.6% (11/56) in Eastern Ethiopia.

**Table 4:** **Distribution of *pfcrt*, *pfmdr1 *** **and *pvmdr1 *** **point mutations in the investigated clinical isolates in Southern and Eastern Ethiopia**

	***P. falciparum***	**SE no**	**SE (%)**	**EE no**	**EE (%)**
*Pfcrt*	only C72	5	2.9%	2	8.0%
	C72 and K76	4	2.4%	1	4.0%
	only K76	23	13.5%	8	32.0%
*pfcrt* and *pfmdr1*	K76 and N86	2	1.2%	2	8.0%
*pfmdr1*	only N86	22	12.9%	7	28.0%
	N86 and Y184	6	3.5%	2	8.0%
	only Y184	7	4.1%	3	12.0%
	*P. vivax*				
*pvmdr1*	only Y976	59	37.1%	11	19.6%
	only F1076	0			0.0%

*pfmdr1*: *Plasmodium falciparum* multi-drug resistance 1; *pfcrt*: *Plasmodium falciparum* chloroquine resistance transporter; *pvmdr1*: *Plasmodium. vivax* multi-drug resistance 1; SE: Southern Ethiopia; EE: Eastern Ethiopia.

The *pfcrt* haplotypes were reconstructed for mutations at codons 72–76 in all *P. falciparum* isolates. The *pfcrt* wild type Cysteine-Valine-Methionine-Asparagine-Lysine (*pfcrt* CVMNK) mutates to either Cysteine-Valine-Isoleucine-Glutamate-Threonine (*pfcrt* CVIET) or Serine-Valine-Methionine-Asparagine-Threonine (*pfcrt* SVMNT) variants Table 5. The mutated *pfcrt* SVMNT haplotype was observed in 4.1% (8/195) of the isolates, whereas *pfcrt* CVMNK of wildtype was observed in 95.9% (187/195) Table 5. No *pfcrt* CVIET haplotypes were observed.

**Table 5:** **Distribution of reconstructed *Pfcrt *** **haplotypes from clinical isolates from South and Eastern Ethiopia**

**Locus**	**haplotype**	**Investigated fragment (5′-3′)**	**Number (%)**
*pfcrt* 74-76	CVMNK (Sensitive)	5′-TAATTGAAACAATTTTTG-3′	187 (95.9)
	SVMNT (Mutant)	5′-TAAT***GA***A***T***A***A***AATTTTTG-3′	8 (4.1)
	CVIET (Mutant)	5′-TAAT***GA***A***T***ACAATTTTTG-3′	0

*pfmdr1*: *Plasmodium falciparum* multi-drug resistance 1; *pfcrt*: *Plasmodium falciparum* chloroquine resistance transporter; *pvmdr1*: *Plasmodium vivax* multi-drug resistance 1.

## Discussion

The aim of the current study was to investigate the prevalence of mutant SNPs in the loci *pfmdr1*, *pfcrt*, and *pvmdr1*, as well as copy number variations in *pfmdr1*, all of which are associated with anti-malarial drug resistance. A large proportion of *P. falciparum* clinical isolates in this study (84.1%) carried the chloroquine-susceptible *pfcrt* K76 allele. Previously it has been reported that the CQ-resistant mutation *pfcrt* K76T is a strong predictor of overall CQ resistance, and thus it is used as a bio-marker of CQ resistance [16]. It appears that CQ-susceptible *Plasmodium falciparum* isolates are back in circulation in Ethiopia.

The finding of this study does not support a previous study conducted in Ethiopia that reported a 100% frequency of the *pfcrt* T76 mutation, and an 81% frequency of the mutation *pfmdr1* Y86 in *P. falciparum*[41]*.* However, the current finding is in line with studies conducted in two East African countries [27,42], where the frequency of the *pfcrt*-76 resistance alleles were repopulated with the sensitive ones after many years of CQ withdrawal. Studies showed that *pfcrt* mutations at codons 72 to 76 with non-synonymous substitutions result in wildtype *pfcrt* CVMNK or other *pfcrt* haplotypes CVIET or SVMNT that have been associated with CQ resistance[43]. The wild type *pfcrt* CVMNK haplotypes were observed in 187 (95.9%) of the *P. falciparum* isolates. The mutant *pfcrt* SVMNT was carried by 4.1% (8/195) of the investigated isolates, and it was identical to that of chloroquine resistant isolates identified in South-East Asia [44]. These are the first findings on the presence of the *pfcrt* SVMNT haplotype in clinical isolates in Ethiopia. This is an interesting finding and novel to the study as the SVMNT haplotype is still very rare in Africa. So far few studies reported the presence of this haplotype in African countries [45-48], of this haplotype mostly associated with amodiaquine resistance and lower level of chloroquine resistance compared to CVIET [49,50]. The *pfcrt* CVIET haplotypes were not detected here, although it is prevalent in Africa [51].

Concerning *pfmdr1*, while wild-type *pfmdr1* is thought to transport and accumulate CQ in the parasites’ food vacuole, mutations N86Y, S1034C, N1042D, and D1246Y interfere with transportation of the anti-malarial drugs, leading to reduced CQ-sensitivity [14]. The percentage of wild-type alleles at codon 86 of *pfmdr1* was 85.1% (166/195), which is higher than 81% present in Schunk in 2006 and 84.5% in Eshetu in 2010 [41,52]. It should be clear that the study by Schunk in 2006 was conducted 7 years before the current study. This is clear that as the time difference between these two studies is large and with reduced drug pressure on CQ over time might have contributed to the return of CQ susceptibility.

The *pfcrt* K76T mutation in Southern Ethiopia was 13.5% (23/170) of the clinical isolates investigated compared to 86.5% (147/170) wild type allele that were sensitive to CQ. The *pfmdr1* locus N86Y was observed in 12.9% (22/170) compared to 87.1% (148/170) wild type allele that were sensitive to CQ. In Eastern Ethiopia, SNPs in the *pfmdr1* locus N86Y and *pfcrt* locus K76T were 28.0% (7/25) and 32% (8/25) respectively. There was no statistical difference between Southern and Eastern Ethiopia in SNPs in the *pfmdr1* locus N86Y and *pfcrt* locus K76T (P = 0.9).

Double mutations in *pvmdr1* at positions Y976F and F1076L have been suggested to be associated with reduced chloroquine susceptibility to *P. vivax* in Thailand and Indonesia, the two countries where *P. vivax* is prevalent. Other studies have addressed whether chloroquine or amodiaquine treatment failure might be associated with *P. vivax* isolates carrying the Y976F substitution [53]. *Pvmdr1* sequences from *P.vivax*, the orthologue of *pfmdr1*, were analyzed for the presence of mutations at positions 976 and 1076, and we found that 32.6% (70/215) of the isolates carried the F976 SNP. Mutations were not detected at codon 1076 in all *P. vivax* isolates studied here. On the other hand, for the investigated *pvmdr1* locus Y976 (Tyr976Phe), the mutation was observed in 37.1% (59/159) of the clinical isolates from Southern Ethiopia compared to 19.6% (11/56) from Eastern Ethiopia.

In line with the current study, studies conducted in Madagascar and Brazil showed that F1076L cannot be taken as an indicator of drug resistance, but rather it is a geographic variant [46,53,54]. Earlier, *in vivo* studies conducted in Ethiopia confirmed the presence of drug resistant *P. vivax*[8,35]. However, these studies were confounded by the inherent difficulties associated with *in vivo* tests for relapsing malaria [55] and the difficulties in differentiating between re-infection, recrudescence, and relapse after treatment failure [56].

A high proportion (97.9%, 191/195) of samples had chloroquine (CQ)-susceptible alleles at both codon 76 of *pfcrt* and codon 86 of *pfmdr1*. The rapid shift in *P. falciparum* from CQ-resistant to CQ-susceptible suggests that the removal of CQ for the treatment of *P. falciparum* or the pressure from AL that has been used since 2004 to treat *falciparum* malaria and mixed infection of *P.falciparum* and *P. vivax* or both may eventually lead to replacement of *pfmdr1* resistance genes by susceptible parasite populations [42,57]. Our finding was consistent with observations from Malawi [57], Kenya [42], and Tanzania [27], where the withdrawal of chloroquine resulted in the rapid spread of a chloroquine-susceptible *Pfcrt* K76 population. In Malawi, recovery of the susceptible *Pfcrt*-K76 from <15% to 100% within 13 years of CQ withdrawal has been reported [57]. Moreover, a study confirmed that the *pfmdr1* alleles 86 N, 184 F, and 1246D significantly increased in prevalence after AL treatment [58]. In another *in vivo* study*, P. falciparum* parasites carrying the chloroquine-susceptible *pfcrt* K76 allele was selected after treatment with AL [59].

It was difficult to determine whether the CQ-susceptible resurgence is due to back-mutations in the CQ-resistant allele or the expansion of surviving CQ-susceptible reservoir populations. Re-expansion appears to be more common in Africa, where transmission rates are higher and naturally immune individuals are more common than in Southeast Asia (where CQ-resistant alleles appear to have gone to fixation in many areas) [57]. It does not appear that CQ-resistant mutants in Ethiopia acquired mutations to compensate for the costs of being drug-resistant. It is believed that such compensatory mutations confer fitness levels greater than that of the original CQ-susceptible and CQ-resistant alleles [60]. CQ-susceptible alleles clearly have a selective advantage over resistant ones, *pfmdr1* Y86 having rebounded from 100% to 11% in 6 years (for samples collected on 2009 from East Ethiopia) to eight years time (for samples collected on 2011from South Ethiopia). On the other hand, N86Y mutation was rebounded from 81% to 9% [41,52].

In Ethiopia, due to resistance developed by *P. falciparum,* replacement of SP by artemether-lumefantrine has been mandatory. Since 2004, artemether-lumefantrine has been the drug of choice for the treatment of *P. falciparum* malaria. Unlike Malawi, in Ethiopia CQ was partially withdrawn and is still the drug of choice for the treatment of uncomplicated *P. vivax*. This finding shows that there has been strong selection for chloroquine-sensitive parasites after the nationwide replacement of chloroquine with SP. The return of chloroquine-susceptible alleles in a country like Ethiopia, which is endemic for malaria, can be considered a positive development toward replacing the expensive artemether-lumefantrine with a safe and cheap form of CQ in combination with other short-acting drugs. Likewise, in malaria-endemic countries even partial returns of chloroquine sensitivity have a positive impact on public health [57].

## Conclusion

The return of CQ-sensitive parasites is likely due to either CQ withdrawal for the treatment of *P. falciparum* or the pressure from AL since 2004 or both. The return of CQ-susceptible *P. falciparum* following declining CQ resistance will have its own contribution in the battle against malaria. If chloroquine susceptibility does become widespread in Ethiopia,the possibility of using chloroquine in the future as a combination therapy with other short-acting drugs with different pharmacokinetic and pharmacodynamics profiles will be an additional anti-malarial option. This is a positive development, since CQ is cheap, safe, well-tolerated, long-lasting, and easy-to-prepare molecule. Additional surveillance studies to investigate resistance markers of *pfmdr1* and *Pfcrt* genes of *P. falciparum* in the same geographic area may help to assess the prevalence of anti-malarial resistance.

## Competing interests

The authors have declared that they have no competing interests.

## Authors’ contributions

SKM designed and performed the field study and experiments, data analysis with drafting first draft. AA,NB, TG,RC, TT and GM contributed to the study design and study samples and revisions of MS. TPV designed the experiments, contributed to materials and tools, supervised the experiments, revision of the MS. All authors read and approved the final manuscript.

## References

[B1] Kebede S Aseffa A Medhin G Berhe N Velavan TP Re-evaluation of microscopy confirmed *Plasmodium falciparum* and *Plasmodium vivax* malaria by nested PCR detection in southern Ethiopia Malar J 2014 13 48 24502664 10.1186/1475-2875-13-48 PMC4011513

[B2] Nevill CG Malaria in sub-Saharan Africa Soc Sci Med 1990 31 667 669 2237509 10.1016/0277-9536(90)90248-q

[B3] O’Meara WP Mangeni JN Steketee R Greenwood B Changes in the burden of malaria in sub-Saharan Africa Lancet Infect Dis 2010 10 545 555 20637696 10.1016/S1473-3099(10)70096-7

[B4] WHO World Malaria Report 2013 Geneva, Switzerland: World Health Organization fact sheet

[B5] Olatunde A Chloroquine-resistant *Plasmodium falciparum* and malaria in Africa Trans R Soc Trop Med Hyg 1977 71 80 81 324040 10.1016/0035-9203(77)90213-9

[B6] Campbell CC Chin W Collins WE Teutsch SM Moss DM Chloroquine-resistant *Plasmodium falciparum* from East Africa: cultivation and drug sensitivity of the Tanzanian I/CDC strain from an American tourist Lancet 1979 2 1151 1154 91887 10.1016/s0140-6736(79)92383-3

[B7] Dondorp AM Nosten F Yi P Das D Phyo AP Tarning J Lwin KM Ariey F Hanpithakpong W Lee SJ Ringwald P Silamut K Imwong M Chotivanich K Lim P Herdman T An SS Yeung S Singhasivanon P Day NP Lindegardh N Socheat D White NJ Artemisinin resistance in *Plasmodium falciparum* malaria N Engl J Med 2009 361 455 467 19641202 10.1056/NEJMoa0808859 PMC3495232

[B8] Tulu AN Webber RH Schellenberg JA Bradley DJ Failure of chloroquine treatment for malaria in the highlands of Ethiopia Trans R Soc Trop Med Hyg 1996 90 556 557 8944273 10.1016/s0035-9203(96)90322-3

[B9] Abebe W Therapeutic efficacy of sulfadoxin/pyrimethamine in the treatment of uncomplicated *Plasmodium falciparum* malaria in Enseno, Meskan Woreda, Gurage zone, SNNPR, Ethiopia Ethiop Med J 2006 44 133 138 17447375

[B10] Gebru-Woldearegai T Hailu A Grobusch MP Kun JF Molecular surveillance of mutations in dihydrofolate reductase and dihydropteroate synthase genes of *Plasmodium falciparum* in Ethiopia Am J Trop Med Hyg 2005 73 1131 1134 16354825

[B11] Degefa T In vivo sulphadoxine-pyrimethamine sentitivity study Tigray Region, Southern Zone, Alamata Town, September–November 2001 Ethiop Med J 2004 42 35 39 15884275

[B12] Jima D Tesfaye G Medhin A Kebede A Argaw D Babaniyi O Efficacy of sulfadoxine-pyrimethamine for the treatment of uncomplicated falciparum malaria in Ethiopia East Afr Med J 2005 82 391 395 16261914 10.4314/eamj.v82i8.9322

[B13] FMOH Malaria Diagnosis and Treatment a Guideline for Health Workers in Ethiopia Ethiopian Federal Ministry of Health Guideline 2004

[B14] Andriantsoanirina V Ratsimbasoa A Bouchier C Tichit M Jahevitra M Rabearimanana S Raherinjafy R Mercereau-Puijalon O Durand R Menard D Chloroquine clinical failures in *P. falciparum* malaria are associated with mutant Pfmdr-1, not Pfcrt in Madagascar PLoS One 2010 5 e13281 20967251 10.1371/journal.pone.0013281 PMC2954150

[B15] Dlamini SV Beshir K Sutherland CJ Markers of anti-malarial drug resistance in *Plasmodium falciparum* isolates from Swaziland: identification of pfmdr1-86 F in natural parasite isolates Malar J 2010 9 68 20199676 10.1186/1475-2875-9-68 PMC2845184

[B16] Griffing S Syphard L Sridaran S McCollum AM Mixson-Hayden T Vinayak S Villegas L Barnwell JW Escalante AA Udhayakumar V pfmdr1 amplification and fixation of pfcrt chloroquine resistance alleles in *Plasmodium falciparum* in Venezuela Antimicrob Agents Chemother 2010 54 1572 1579 20145087 10.1128/AAC.01243-09 PMC2849383

[B17] Wurtz N Fall B Pascual A Diawara S Sow K Baret E Diatta B Fall KB Mbaye PS Fall F Dieme Y Rogier C Bercion R Briolant S Wade B Pradines B Prevalence of molecular markers of *Plasmodium falciparum* drug resistance in Dakar, Senegal Malar J 2012 11 197 22694921 10.1186/1475-2875-11-197 PMC3470961

[B18] Baro NK Callaghan PS Roepe PD Function of resistance conferring *Plasmodium falciparum* chloroquine resistance transporter isoforms Biochemistry 2013 52 4242 4249 23688277 10.1021/bi400557x PMC3703759

[B19] Fidock DA Nomura T Talley AK Cooper RA Dzekunov SM Ferdig MT Ursos LM Sidhu AB Naude B Deitsch KW Su XZ Wootton JC Roepe PD Wellems TE Mutations in the *P. falciparum* digestive vacuole transmembrane protein PfCRT and evidence for their role in chloroquine resistance Mol Cell 2000 6 861 871 11090624 10.1016/s1097-2765(05)00077-8 PMC2944663

[B20] Norahmad NA Abdullah NR Yaccob N Jelip J Dony JF Ruslan KF Sulaiman LH Sidek HM Noedl H Ismail Z High prevalence of pfcrt K76t mutants among *Plasmodium falciparum* isolates from Sabah, Malaysia Southeast Asian J Trop Med Public Health 2011 42 1322 1326 22299399

[B21] Sanchez CP McLean JE Rohrbach P Fidock DA Stein WD Lanzer M Evidence for a pfcrt-associated chloroquine efflux system in the human malarial parasite *Plasmodium falciparum* Biochemistry 2005 44 9862 9870 16026158 10.1021/bi050061f

[B22] Brega S Meslin B De Monbrison F Severini C Gradoni L Udomsangpetch R Sutanto I Peyron F Picot S Identification of the *Plasmodium vivax* mdr-like gene (pvmdr1) and analysis of single-nucleotide polymorphisms among isolates from different areas of endemicity J Infect Dis 2005 191 272 277 15609238 10.1086/426830

[B23] Mula P Fernandez-Martinez A de Lucio A Ramos JM Reyes F Gonzalez V Benito A Berzosa P Detection of high levels of mutations involved in anti-malarial drug resistance in Plasmodium falciparum and Plasmodium vivax at a rural hospital in southern Ethiopia Malar J 2011 10 214 21810256 10.1186/1475-2875-10-214 PMC3161020

[B24] Duraisingh MT Cowman AF Contribution of the pfmdr1 gene to antimalarial drug-resistance Acta Trop 2005 94 181 190 15876420 10.1016/j.actatropica.2005.04.008

[B25] Valderramos SG Fidock DA Transporters involved in resistance to antimalarial drugs Trends Pharmacol Sci 2006 27 594 601 16996622 10.1016/j.tips.2006.09.005 PMC2944664

[B26] Picot S Olliaro P De Monbrison F Bienvenu AL Price RN Ringwald P A systematic review and meta-analysis of evidence for correlation between molecular markers of parasite resistance and treatment outcome in falciparum malaria Malar J 2009 8 89 19413906 10.1186/1475-2875-8-89 PMC2681474

[B27] Mohammed A Ndaro A Kalinga A Manjurano A Mosha JF Mosha DF Van Zwetselaar M Koenderink JB Mosha FW Alifrangis M Reyburn H Roper C Kavishe RA Trends in chloroquine resistance marker, Pfcrt-K76T mutation ten years after chloroquine withdrawal in Tanzania Malar J 2013 12 415 24225406 10.1186/1475-2875-12-415 PMC3830541

[B28] Vathsala PG Pramanik A Dhanasekaran S Devi CU Pillai CR Subbarao SK Ghosh SK Tiwari SN Sathyanarayan TS Deshpande PR Mishra GC Ranjit MR Dash AP Rangarajan PN Padmanaban G Widespread occurrence of the *Plasmodium falciparum* chloroquine resistance transporter (Pfcrt) gene haplotype SVMNT in *P. falciparum* malaria in India Am J Trop Med Hyg 2004 70 256 259 15031513

[B29] Price RN Uhlemann AC Brockman A McGready R Ashley E Phaipun L Patel R Laing K Looareesuwan S White NJ Nosten F Krishna S Mefloquine resistance in *Plasmodium falciparum* and increased pfmdr1 gene copy number Lancet 2004 364 438 447 15288742 10.1016/S0140-6736(04)16767-6 PMC4337987

[B30] Ballut PC Siqueira AM Orlando AC Alexandre MA Alecrim MG Lacerda MV Prevalence and risk factors associated to pruritus in *Plasmodium vivax* patients using chloroquine in the Brazilian Amazon Acta Trop 2013 128 504 508 23906614 10.1016/j.actatropica.2013.07.008

[B31] Galappaththy GN Tharyan P Kirubakaran R Primaquine for preventing relapse in people with *Plasmodium vivax* malaria treated with chloroquine Cochrane Database Syst Rev 2013 10 CD004389 10.1002/14651858.CD004389.pub3 PMC6532739 24163057

[B32] Ganguly S Saha P Guha SK Das S Bera DK Biswas A Kundu PK Saha B Ray K Maji AK In vivo therapeutic efficacy of chloroquine alone or in combination with primaquine against vivax malaria in Kolkata, West Bengal, India, and polymorphism in pvmdr1 and pvcrt-o genes Antimicrob Agents Chemother 2013 57 1246 1251 23262997 10.1128/AAC.02050-12 PMC3591881

[B33] Rieckmann KH Davis DR Hutton DC *Plasmodium vivax* resistance to chloroquine? Lancet 1989 2 1183 1184 2572903 10.1016/s0140-6736(89)91792-3

[B34] Baird JK Sustriayu Nalim MF Basri H Masbar S Leksana B Tjitra E Dewi RM Khairani M Wignall FS Survey of resistance to chloroquine by *Plasmodium vivax* in Indonesia Trans R Soc Trop Med Hyg 1996 90 409 411 8882190 10.1016/s0035-9203(96)90526-x

[B35] Teka H Petros B Yamuah L Tesfaye G Elhassan I Muchohi S Kokwaro G Aseffa A Engers H Chloroquine-resistant *Plasmodium vivax* malaria in Debre Zeit, Ethiopia Malar J 2008 7 220 18959774 10.1186/1475-2875-7-220 PMC2584068

[B36] Vestergaard LS Ringwald P Responding to the challenge of antimalarial drug resistance by routine monitoring to update national malaria treatment policies Am J Trop Med Hyg 2007 77 153 159 18165488

[B37] Snounou G Beck HP The use of PCR genotyping in the assessment of recrudescence or reinfection after antimalarial drug treatment Parasitol Today 1998 14 462 467 17040849 10.1016/s0169-4758(98)01340-4

[B38] Humphreys GS Merinopoulos I Ahmed J Whitty CJ Mutabingwa TK Sutherland CJ Hallett RL Amodiaquine and artemether-lumefantrine select distinct alleles of the *Plasmodium falciparum* mdr1 gene in Tanzanian children treated for uncomplicated malaria Antimicrob Agents Chemother 2007 51 991 997 17194834 10.1128/AAC.00875-06 PMC1803116

[B39] Ferreira ID Rosario VE Cravo PV Real-time quantitative PCR with SYBR Green I detection for estimating copy numbers of nine drug resistance candidate genes in P*lasmodium falciparum* Malar J 2006 5 1 16420686 10.1186/1475-2875-5-1 PMC1363351

[B40] Livak KJ Schmittgen TD Analysis of relative gene expression data using real-time quantitative PCR and the 2(-Delta Delta C(T)) Method Methods 2001 25 402 408 11846609 10.1006/meth.2001.1262

[B41] Schunk M Kumma WP Miranda IB Osman ME Roewer S Alano A Loscher T Bienzle U Mockenhaupt FP High prevalence of drug-resistance mutations in *Plasmodium falciparum* and *Plasmodium vivax* in southern Ethiopia Malar J 2006 5 54 16817953 10.1186/1475-2875-5-54 PMC1524791

[B42] Mang’era CM Mbai FN Omedo IA Mireji PO Omar SA Changes in genotypes of *Plasmodium falciparum* human malaria parasite following withdrawal of chloroquine in Tiwi, Kenya Acta Trop 2012 123 202 207 22641431 10.1016/j.actatropica.2012.05.007

[B43] Dittrich S Alifrangis M Stohrer JM Thongpaseuth V Vanisaveth V Phetsouvanh R Phompida S Khalil IF Jelinek T Falciparum malaria in the north of Laos: the occurrence and implications of the *Plasmodium falciparum* chloroquine resistance transporter (pfcrt) gene haplotype SVMNT Trop Med Int Health 2005 10 1267 1270 16359407 10.1111/j.1365-3156.2005.01514.x

[B44] Awasthi G Prasad GB Das A Population genetic analyses of *Plasmodium falciparum* chloroquine receptor transporter gene haplotypes reveal the evolutionary history of chloroquine-resistant malaria in India Int J Parasitol 2011 41 705 709 21447338 10.1016/j.ijpara.2011.03.002

[B45] Alifrangis M Dalgaard MB Lusingu JP Vestergaard LS Staalsoe T Jensen AT Enevold A Ronn AM Khalil IF Warhurst DC Lemnge MM Theander TG Bygbjerg IC Occurrence of the Southeast Asian/South American SVMNT haplotype of the chloroquine-resistance transporter gene in *Plasmodium falciparum* in Tanzania J Infect Dis 2006 193 1738 1741 16703518 10.1086/504269

[B46] Gama BE Oliveira NK Souza JM Daniel-Ribeiro CT Ferreira-da-Cruz MF Characterisation of pvmdr1 and pvdhfr genes associated with chemoresistance in Brazilian *Plasmodium vivax* isolates Mem Inst Oswaldo Cruz 2009 104 1009 1011 20027469 10.1590/s0074-02762009000700012

[B47] Gbotosho GO Folarin OA Bustamante C Da Silva LH Mesquita E Sowunmi A Zalis MG Oduola AM Happi CT Different patterns of pfcrt and pfmdr1 polymorphisms in P. falciparum isolates from Nigeria and Brazil: the potential role of antimalarial drug selection pressure Am J Trop Med Hyg 2012 86 211 213 22302850 10.4269/ajtmh.2012.11-0368 PMC3269269

[B48] Mehlotra RK Mattera G Bockarie MJ Maguire JD Baird JK Sharma YD Alifrangis M Dorsey G Rosenthal PJ Fryauff DJ Kazura JW Stoneking M Zimmerman PA Discordant patterns of genetic variation at two chloroquine resistance loci in worldwide populations of the malaria parasite *Plasmodium falciparum* Antimicrob Agents Chemother 2008 52 2212 2222 18411325 10.1128/AAC.00089-08 PMC2415780

[B49] Sa JM Twu O Hayton K Reyes S Fay MP Ringwald P Wellems TE Geographic patterns of *Plasmodium falciparum* drug resistance distinguished by differential responses to amodiaquine and chloroquine Proc Natl Acad Sci U S A 2009 106 18883 18889 19884511 10.1073/pnas.0911317106 PMC2771746

[B50] Sa JM Twu O Protecting the malaria drug arsenal: halting the rise and spread of amodiaquine resistance by monitoring the PfCRT SVMNT type Malar J 2010 9 374 21182787 10.1186/1475-2875-9-374 PMC3020158

[B51] Menard S Morlais I Tahar R Sayang C Mayengue PI Iriart X Benoit-Vical F Lemen B Magnaval JF Awono-Ambene P Basco LK Berry A Molecular monitoring of *Plasmodium falciparum* drug susceptibility at the time of the introduction of artemisinin-based combination therapy in Yaounde, Cameroon: implications for the future Malar J 2012 11 113 22498364 10.1186/1475-2875-11-113 PMC3368752

[B52] Eshetu T Berens-Riha N Fekadu S Tadesse Z Gurkov R Holscher M Loscher T Miranda IB Different mutation patterns of *Plasmodium falciparum* among patients in Jimma University Hospital, Ethiopia Malar J 2010 9 226 20691106 10.1186/1475-2875-9-226 PMC2922303

[B53] Marfurt J De Monbrison F Brega S Barbollat L Muller I Sie A Goroti M Reeder JC Beck HP Picot S Genton B Molecular markers of in vivo *Plasmodium vivax* resistance to amodiaquine plus sulfadoxine-pyrimethamine: mutations in pvdhfr and pvmdr1 J Infect Dis 2008 198 409 417 18582193 10.1086/589882

[B54] Barnadas C Ratsimbasoa A Tichit M Bouchier C Jahevitra M Picot S Menard D *Plasmodium vivax* resistance to chloroquine in Madagascar: clinical efficacy and polymorphisms in pvmdr1 and pvcrt-o genes Antimicrob Agents Chemother 2008 52 4233 4240 18809933 10.1128/AAC.00578-08 PMC2592859

[B55] Baird JK Leksana B Masbar S Fryauff DJ Sutanihardja MA Suradi Wignall FS Hoffman SL Diagnosis of resistance to chloroquine by *Plasmodium vivax*: timing of recurrence and whole blood chloroquine levels Am J Trop Med Hyg 1997 56 621 626 9230792 10.4269/ajtmh.1997.56.621

[B56] Chen N Auliff A Rieckmann K Gatton M Cheng Q Relapses of *Plasmodium vivax* infection result from clonal hypnozoites activated at predetermined intervals J Infect Dis 2007 195 934 941 17330782 10.1086/512242

[B57] Laufer MK Takala-Harrison S Dzinjalamala FK Stine OC Taylor TE Plowe CV Return of chloroquine-susceptible falciparum malaria in Malawi was a reexpansion of diverse susceptible parasites J Infect Dis 2010 202 801 808 20662717 10.1086/655659 PMC3380613

[B58] Dokomajilar C Nsobya SL Greenhouse B Rosenthal PJ Dorsey G Selection of *Plasmodium falciparum* pfmdr1 alleles following therapy with artemether-lumefantrine in an area of Uganda where malaria is highly endemic Antimicrob Agents Chemother 2006 50 1893 1895 16641472 10.1128/AAC.50.5.1893-1895.2006 PMC1472234

[B59] Sisowath C Petersen I Veiga MI Martensson A Premji Z Bjorkman A Fidock DA Gil JP In vivo selection of *Plasmodium falciparum* parasites carrying the chloroquine-susceptible pfcrt K76 allele after treatment with artemether-lumefantrine in Africa J Infect Dis 2009 199 750 757 19210165 10.1086/596738 PMC2718568

[B60] Levin BR Perrot V Walker N Compensatory mutations, antibiotic resistance and the population genetics of adaptive evolution in bacteria Genetics 2000 154 985 997 10757748 10.1093/genetics/154.3.985 PMC1460977

